# Geographic patterns of hepatocellular carcinoma mortality with exposure to iron in groundwater in Taiwanese population: An ecological study

**DOI:** 10.1186/1471-2458-13-352

**Published:** 2013-04-16

**Authors:** Horng-Jeng Shyu, Chia-Chi Lung, Chien-Chang Ho, Yi-Hua Iris Sun, Pei-Chieh Ko, Jing-Yang Huang, Chia-Chen Pan, Yi-Chen Chiang, Shih-Chang Chen, Yung-Po Liaw

**Affiliations:** 1 Jen-Ai Hospital, Taichung City 41265, Taiwan; 2 Department of Healthcare Administration, Asia University, Taichung City 41354, Taiwan; 3 Department of Public Health and Institute of Public Health, Chung Shan Medical University, Taichung City 40201, Taiwan; 4 Department of Family and Community Medicine, Chung Shan Medical University Hospital, Taichung 40201, Taiwan; 5 Department of Health and Leisure Management, Yuanpei University, Hsinchu City 30015, Taiwan; 6 School of Dentistry, Chung Shan Medical University, Taichung City 40201, Taiwan; 7 Department of Leisure Industry and Health Promotion, National Ilan University, Yilan County 26047, Taiwan; 8 Department of Public Health and Institute of Public Health, Chung Shan Medical University, No. 110, Sec. 1, Chien-Kuo N. Road, Taichung City 40201, Taiwan

**Keywords:** Hepatocellular carcinoma, Age-standardized mortality, Land subsidence, Iron, Groundwater, Geographical information systems

## Abstract

**Background:**

Many studies have examined the risk factors for HCC (including hepatitis B virus, hepatitis C virus, aflatoxin, retinol, cigarette smoking, and alcohol consumption). However, data from previous studies on the association between iron exposure, land subsidence, and HCC mortality/incidence were limited, especially in Taiwanese population. We aimed to explore the geographical distribution of HCC mortality rates by township-specific data and to evaluate the association between HCC mortality, land subsidence, and iron levels in groundwater in Taiwan.

**Methods:**

We conducted an ecological study and calculated the HCC age-standardized mortality/incidence rates according to death certificates issued in Taiwan from 1992 to 2001 and incidence data from 1995–1998. The land subsidence dataset before 2005 and iron concentrations in groundwater in 1989 are also involved in this study. Both geographical information systems and Pearson correlation coefficients were used to analyze the relationship between HCC mortality rates, land subsidence, and iron concentrations in groundwater.

**Results:**

Township-specific HCC mortality rates are higher in southwestern coastal townships where serious land subsidence and higher township-specific concentrations of iron in groundwater are present. The Pearson correlation coefficients of iron concentrations in groundwater and ASRs of HCC were 0.286 (*P* = 0.004) in males and 0.192 (*P* = 0.058) in females for mortality data; the coefficients were 0.375 (*P* < 0.001) in males and 0.210 (*P* = 0.038) in females for incidence data.

**Conclusions:**

This study showed that HCC mortality is clustered in southwestern Taiwan and the association with the iron levels in groundwater in Taiwanese population warrant further investigation.

## Background

Hepatocellular carcinoma (HCC) is the leading cause of death from cancer in males and the second leading cause in females in Taiwan [1]. Hepatitis B virus (HBV) is the well known risk factor for HCC [2]. There are more than 350 million chronic HBV carriers in the world and 75% living in the Asia Pacific regions including Taiwan [3]. A hepatitis B vaccination program was launched in Taiwan since 1984, and it has effectively reduced the prevalence of HBV infection, chronic HBV infection rate, and incidence of HCC in children [4,5]. In addition to children, a 20 year follow-up study has also provided evidence that HCC incidence rate is decreased by the HBV vaccine program [6]. However, to date, the incidence and mortality of HCC among overall Taiwanese population are still regarded as high.

From public health viewpoint, an immediate challenge in cancer prevention and control is to reduce the cancer incidence rate of people living in high-risk areas of developing cancer. For this purpose, the acts of detecting a high-mortality cluster and suggesting possible prevention strategies in addition to HBV vaccination have gained considerable importance. In Taiwan, in addition to HBV and hepatitis C virus (HCV) infections being two dominant factors, other major factors associated with an increased HCC risk include aflatoxin exposure, low consumption of vegetables, low serum levels of retinol, cigarette smoking, alcohol consumption, and iron overload [7-13]. The major factor besides HBV and HCV infections that should be selected for formulating a prevention strategy to lower the incidence of HCC in Taiwan remains a question. Regarding to this, the geographical distribution of cancer provides valuable clues for studying its etiology and helping propose hypotheses that can be tested in analytical studies [14,15]. In one of previous studies, an atlas of cancer mortality in Taiwan from 1968 to 1976 led to a series of comprehensive cancer epidemiology studies in an endemic area of Blackfoot disease. A dose–response relationship between high-arsenic artesian well water and internal organ cancers was documented [16]. Therefore, to prevent HCC in Taiwan, we aimed to explore the geographical distribution of HCC mortality based on township-specific base by evaluating the association between HCC mortality, land subsidence, and exposure to iron in groundwater in Taiwan.

## Methods

### Data collection

The number of certified deaths of HCC stratified by sex and 5-year age groups was published annually from 1992 to 2001 by the Department of Health of Taiwan according to the ninth revision of the International Classification of Diseases (ICD-9). Death as a result of HCC was defined according to ICD-9 code 155. Age-standardized rates (ASRs) were calculated by adopting the direct method in 1976 World Standard Population [17] in 5-year groups of 0 to 4, 5 to 9, 10 to 14, . . . , 80 to 84, and 85 years or more. ASRs are expressed as the average number of annual deaths per 100,000 person years. Similarly, the HCC incidence rate was also computed from 1995–1998 incidence data by cancer registry system.

In order to illustrate and compare the ASRs of HCC in the study areas by sex, different colors were applied to denote the groups of the rates ranked by percentile. The results of this comparison of the township-specific ASRs to the overall ASR in Taiwan and the ranking of the township ASRs were present in following colors with specific indications:

• Red: the highest 10% of all township ASRs in Taiwan and significantly higher than the overall ASR in Taiwan.

• Purple: not among the highest 10% of all township ASRs in Taiwan but significantly higher than the overall ASR in Taiwan.

• Orange: among the highest 10% of all township ASRs in Taiwan but not significantly higher than the overall ASR in Taiwan.

• Green: within 10–90% of all township ASRs in Taiwan and not significantly different with the overall ASR in Taiwan.

• Gray: among the lowest 10% of all township ASRs in Taiwan but not significantly lower than the overall ASR in Taiwan.

• Yellow: not in the lowest 10% of all township ASRs in Taiwan but significantly lower than the overall ASR in Taiwan.

• White: among the lowest 10% of all township ASRs in Taiwan and significantly lower than the overall ASR in Taiwan.

### Land subsidence data

Land subsidence dataset was collected from Water Resources Agency, Ministry of Economic Affairs, Taiwan. Townships having serious land subsidence are defined by the following conditions: region’s ground level was lower than the township’s highest tide level and seawater intrusion occurred frequently; more than 70% of township’s region had an accumulative subsidence of the land surface equal to 50 cm or above from the date of begin monitored to year 2005; subsidence rate was more than 10 cm per year. The land subsidences of townships are classified into three levels: “all region”, “non-all region” and “no region” with serious land subsidence.

### Assessment of exposures to iron in groundwater

Data of untreated tap groundwater consist of water temperature, turbidity, alkalinity, PH value, chloride level, sulfate level, concentrations of metals … etc., provided by Taiwan Water Supply Corporation, by whom the daily average values was measured in 1989.

### Statistical analysis

Township-specific ASRs of HCC were generated by using SAS Software Package (Version 9.12; SAS Institute Inc, Cary, NC); township-specific cancer mortality ASRs, serious land subsidence maps, and iron levels in groundwater were prepared using Geographical Information System (GIS) tool (ArcGIS 9; ESRI, CA, USA). After mapping the geographical variation in HCC mortality, a preliminary study of the hot spots in the geographical distribution was conducted to enable the generation of potential etiological hypotheses. We used Pearson correlation and partial correlation coefficients to evaluate the relationship between township-specific HCC mortality, incidence ASRs and the concentrations of iron in groundwater. In all analyses, values of *P* < 0.05 were considered statistically significant.

## Results

**Figure 1:**
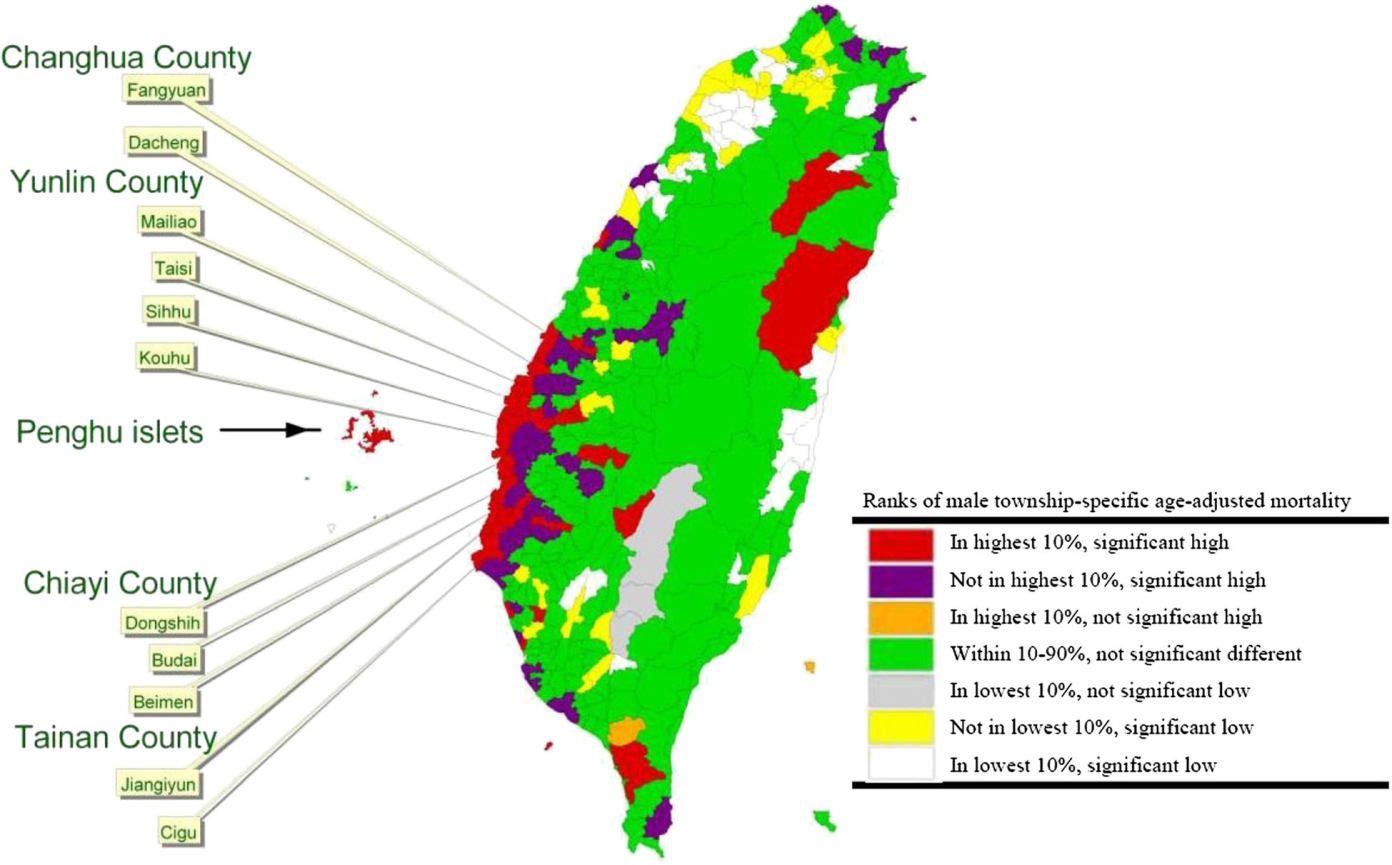
Mortality maps in males based on the township-specific ASRs of HCC by rank in Taiwan during 1992–2001.

**Figure 2:**
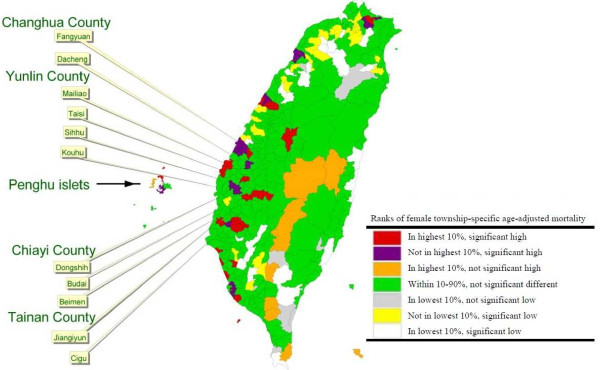
Mortality maps in females based on the township-specific ASRs of HCC by rank in Taiwan during 1992–2001.

Male and female distributions of liver cancer mortality ASRs from 1992 to 2001 ranked by percentile are shown in Figures 1 and 2, respectively. The most striking cluster was being observed on the southwestern coast of Taiwan, and a marked coastal gradient pattern is shown for men; however, no such trend was observed for women. The apparent highest rate per 100,000 person years in the red category for males was observed in the northern and southern regions of the western coastal townships including the following: Fangyuan (ASR = 60.82) and Dacheng (ASR = 60.89) townships in Changhua County; Mailiao (ASR = 80.43), Taisi (ASR = 83.47), Sihhu (ASR = 69.30), and Kouhu (ASR = 65.42) townships in Yunlin County; Dongshih (ASR = 60.34) and Budai (ASR = 61.97) townships in Chiayi County; Beimen (ASR = 66.61), Jiangiyun (ASR = 73.04), and Cigu (ASR = 63.52) townships in Tainan County. For females, the mortality rate per 100,000 person years was 17.67 for Fangyuan and 15.1 for Dacheng in Changhua County; 19.74 for Mailiao, 20.14 for Taisi, 16.03 for Sihhu, and 17.25 for Kouhu in Yunlin County; 10.89 for Dongshih and 9.82 for Budai in Chiayi County; 15.35 for Beimen, 12.88 for Jiangiyun, and 14.61 for Cigu in Tainan County. All those coastal townships with red hot spots are located in the Changhua, Yunlin, Chiayi, and Tainan counties where the largest agricultural county cluster is hosted. Those townships are simultaneously the area with the most serious land subsidence in Taiwan.

**Figure 3:**
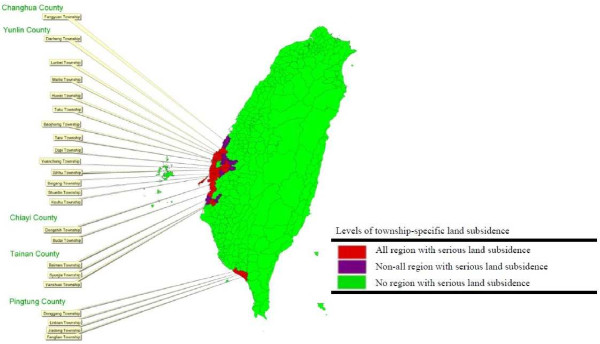
Townships with serious land subsidence located in Changhua, Yunlin, Chiayi, Tainan, and Pingtung counties in Taiwan before 2005.

Figure 3 shows the distribution of townships with serious land subsidence. The red and purple areas indicate the townships having “all” and “non-all” region with serious land subsidence respectively while green areas are townships with no serious land subsidence. Most of townships with serious land subsidence are located in southwestern coastal areas of Taiwan.

**Figure 4:**
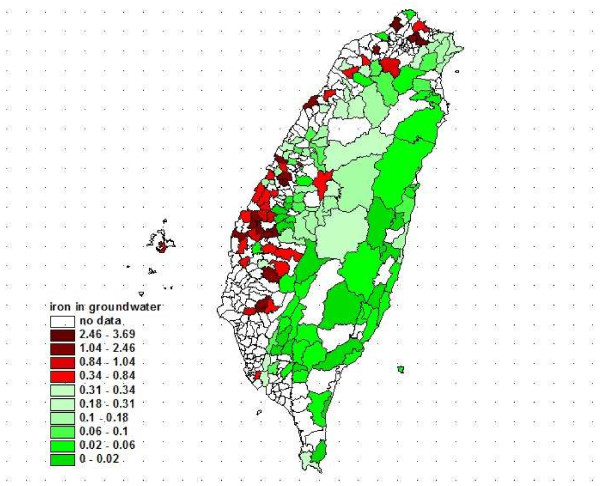
The distribution of iron levels in groundwater from Taiwan in 1989.

The distribution of iron levels in groundwater in 1989 was shown in Figure 4. The most striking cluster was being observed on the southwestern coast of Taiwan, and the pattern is similar with male HCC mortality map.

Table 1 shows concentrations of iron in groundwater and HCC mortality/incidence ASRs in different types of area. Townships with serious land subsidence have significant higher iron concentration and HCC mortality/incidence ASRs than those without serious land subsidence. Table 2 gives the Pearson correlation coefficients of iron concentrations in groundwater and mortality and incidence ASRs for HCC. The HCC mortality ASRs were 0.286 (*P* = 0.004) in males and 0.192 (*P* = 0.058) in females; the HCC incidence ASRs were 0.375 (*P* < 0.001) in males and 0.210 (*P* = 0.038) in females. With the adjustment on land subsidence level, the Pearson partial correlation coefficients of iron concentration in groundwater and mortality/ incidence ASRs for HCC were shown in Table 3. The Pearson partial correlation coefficient in mortality ASRs is 0.217 (*P* = 0.006) for male and 0.128 (*P* = 0.110) for female; and in incidence ASRs is 0.327 (*P* < 0.001) for male and 0.225 (*P* = 0.005) for female.

**Table 1:** **Concentrations of iron in groundwater and HCC mortality and incidence ASRs by land subsidence levels**^**1**^

		**Serious land subsidence**	**No serious land subsidence**	***P*-value**
Iron (mg/l)		1.04 ± 0.20	0.34 ± 0.05	< 0.001
HCC Mortality ASRs	Male	61.35 ± 3.13	37.78 ± 1.05	< 0.001
	Female	16.86 ± 1.81	11.26 ± 0.37	< 0.001
HCC Incidence ASRs	Male	62.11 ± 5.18	34.46 ± 1.29	< 0.001
	Female	15.24 ± 1.67	11.01 ± 0.57	< 0.001

^1^Township-specific concentrations of iron in groundwater are average values of daily measured in 1989; Township-specific HCC incidence and mortality ASRs during 1992–2001 are direct age-adjusted rates by 1979 WHO.

**Table 2:** Pearson correlation coefficient between concentrations of iron in groundwater (mg/l) and HCC mortality and incidence ASRs (per 100,000 person years)

	**Iron concentration**	
	**Correlation coefficient**	***P*-value**
HCC mortality ASRs		
Male	0.286	0.004^**^
Female	0.192	0.058
HCC incidence ASRs		
Male	0.375	< 0.001^***^
Female	0.210	0.038^*^

*P < 0.05; **P < 0.01; ***P < 0.001.

**Table 3:** **Pearson partial correlation coefficient between concentrations of iron in groundwater and HCC mortality and incidence ASRs**^**1**^

		**Concentrations of iron in groundwater**	
		**Correlation coefficient**	***P*-value**
HCC mortality ASRs	Male	0.217	0.006**
	Female	0.128	0.110
HCC incidence ASRs	Male	0.327	< 0.001***
	Female	0.225	0.005**

^1^Adjusted for land subsidence levels.

*P < 0.05; **P < 0.01; ***P < 0.001.

## Discussion

Experience suggests that aggregations of cancer incidences on a small-area scale may provide clues to identify environmental or lifestyle risk factors. As what is expected, it is important to take notice of fluctuations in diagnosis, reporting, survival time, migration, and mobility. Since every sociodemographical event including birth, death, and migration is mandatorily registered in Taiwan, the demographical data reported by the Household Registration Offices in Taiwan are essentially complete and accurate. In Taiwan, it is mandatory to submit death certificates to household registration offices, the death certificate registry in Taiwan is basically complete. We showed the maps of ranks township-specific age-adjusted mortality rate. We found that the age-adjusted mortality rates were significantly higher in townships on the southwestern coast than those in other townships. The difference of mortality rates between southwestern coastal townships and other townships could not merely result from a variation in survival times because the overall five-year survival rate for liver cancer in Taiwan was 15% during 1987–1992 [18]. Although the association between concentrations of iron in groundwater and HCC mortality rate did not reach statistical significance for females (*P* = 0.058), a statistical significance did exist for both genders in the correlation coefficient between concentrations of iron in groundwater and HCC incidence rates. The correlation coefficients were less affected (in comparison with those for mortality data) by the adjustment of land subsidence. In particular, after adjusting for land subsidence, the correlation coefficient for mortality in women was not significant and very close to the null value (0.128 only). On the other hand, after adjusting for land subsidence, the correlation coefficients for incidence data had less reduction in the absolute values and remained statistically significant. It seems that the incidence data had more stable correlations with the iron levels.

Why did the most striking cluster of mortality ASRs of HCC in males occur in the southwestern coastal regions (Changhua, Yunlin, Chiayi, and Tainan county) of Taiwan? A series of epidemiological studies on major risk factors for liver cancer in Taiwan have been carried out. Both HBV and HCV infections are major risk factors for HCC in Taiwan [7,8,19-24]. On the other hand, previous studies have demonstrated that the mortality rate of liver cancer is strikingly high in the Matzu islets (located near the north coast of Fukien Province of mainland China and separated from Taiwan Island by the Taiwan Strait), Penghu islets, and the endemic area of arseniasis in southwestern Taiwan; however, the seroprevalence of HBsAg among the residents of these areas was similar or slightly higher than that among general Taiwanese population [19,25-27]. Moreover, the seroprevalence of anti-HCV antibodies in Matzu was even lower than that among general Taiwanese population [19]. These results suggest that major risk factors in addition to HBV and HCV infections can be involved in the etiology of liver cancer, and those factors are responsible for the high liver cancer mortality in the Matzu and Penghu islets and in southwestern Taiwan. Chen et al. [28] revealed a geographic variation in HBV and HCV seroprevalence in Taiwan from a large-scale survey of free hepatitis screening participants. It was found that the highest anti-HCV positive rates were in Miaoli County, Chiayi County, Chiayi City, and Yunlin County while the highest HBsAg positive rates were in Keelung City and Yilan City. That is, the areas with higher HBV or HCV prevalence rate are not clustering at the areas with land subsidence, but the higher HCC mortality rates were clustered at the land subsidence. These indicate that HBV and HCV infection aren’t the only risk factors that influence the incidence of HCC.

What is the possible major risk factor besides HBV and HCV infections that involved in the etiology of liver cancer and is responsible for the most striking geographical cluster observed in southwestern Taiwan? The fact that the incidence of HCC varies greatly around the world suggests the involvement of environmental etiological factors. Some studies showed that excess uptake of iron is possibly involved in carcinogenesis [29,30]. The experimental studies have also supported the hypothesis that iron may facilitate the development of HCC in cirrhotic and non-cirrhotic patients [31]. Furthermore, long-term treatment of patients with chronic HCV infection with iron-reducing agents results in a significant improvement of liver function and marked reduction in the progression to HCC [32,33]. In another study [34], iron deposition was found in 43% of the HCC cases and indicated that, in Myanmar patients with HCC, iron deposition might accelerate hepatocarcinogenesis, by promoting cancer cell proliferation, without affecting the Fas/FasL apoptotic system. A recently study [35] showed that chronic iron overload seemed to induce nuclear localization of MT and NF-jB activation, with a resultant marked acceleration of liver regeneration in ironoverload rats after PH occurring at least 12 h earlier than that in normal-diet rats. The accelerated liver regeneration was likely due to the shortening of G0–G1 transition, possibly by bypassing the signaling cascades required for the induction of hepatocyte proliferation. This accelerated response by chronic iron overload could be involved in the promotion of hepatocellular carcinogenesis under infection with hepatitis viruses. From epidemiology viewpoint, the interaction between human uptake of overload iron and HBV/HCV infection may play an important role on the incidence of HCC which worths a further study.

Groundwater is a major source of water supply in southwestern coastal Taiwan due to the shortage of surface water mainly obtained from a few creeks and rivers. The over-pumping of groundwater for aquaculture has caused serious land subsidence in southwestern coastal Taiwan [36]. Serious land subsidence areas are located in the counties of Changhua and Yunlin, the southwestern part of the alluvial fan of Chou-Shui River (Figure 3), where the supply rates of tap water from surface water in Changhua and Yunlin are the lowest among all the counties in Taiwan (less than 36.95% and 52.51% in 1975 and 1981 respectively). On the other hand, the iron concentrations of untreated tap water pumped from groundwater in “serious land subsidence areas” and “no serious land subsidence areas” were found to be 1.04 (± 0.20) and 0.34 (± 0.05) mg/l respectively (Table 1). According to a WHO report, the concentrations of iron in drinking water are normally less than 0.3 mg/l [37]. The above statements suggest that the residents of the serious land subsidence areas have been consuming a large quantity of groundwater with high iron concentration for drinking and water supplies for aquaculture and fishponds over several decades. Table 1 shows that, in both males and females, the average ASRs of HCC among the residents of serious land subsidence areas were significantly higher than ASRs among areas of no serious land subsidence.

Unlike arsenic, a confirmed human carcinogen, the carcinogenicity of iron remains debatable. However, increasing evidence summarized below shows that iron overload can contribute to cancer development either as an initiator or a promoter [10]. The toxicity of iron is largely based on Fenton and Haber-Weiss chemistry wherein catalytic amounts of iron are sufficient to yield hydroxyl radicals (OH.) from superoxide (O_2_^.–^) and hydrogen peroxide (H_2_O_2_), collectively known as “reactive oxygen intermediates” (ROIs). Importantly, ROIs are inevitable byproducts of aerobic respiration and are generated by incomplete reduction of dioxygen in mitochondria [38,39].

Hepatocellular carcinoma patients may have higher iron stores than those who do not develop HCC [40]. Iron can catalyze the production of oxygen radicals that may be proximate carcinogens [39,41]. The results of a study conducted in Taiwan by Stevens et al. [42] were consistent with the hypothesis that increased iron stores increase the risk of primary hepatocellular carcinoma. Moreover, serum ferritin was thought to be related to body iron stores [43,44]. The serum ferritin concentration, which is widely used clinically as an index of body iron stores, was predominantly varying by sex, blood donation, and age. The serum ferritin concentration tends to be lower among women and regular blood donors [45,46], and it can also be expected to run parallel with physiological changes of human body. For example, female serum ferritin is increased before menopause to after menopause [47], but the concentration in females remains lower than that of men until after menopause [47,48]. To sum, this difference in body iron stores is responsible for the gender difference in HCC mortality rate clustering in southwestern Taiwan.

To the best of our knowledge, this study was the first ecologic epidemiology research to examine the association of HCC mortality with the concentration of iron in groundwater in Taiwan. Therefore, there is not yet any evidence to support the correlation of iron deposit in HCC patients with the concentration of iron in groundwater would be indicated in the previous studies. In an excellent work from Soe et al. [34] in Japan, Prussian blue staining was performed in order to detect iron deposition, and the iron deposition was found in 43% of the HCC cases. The study showed that, in Myanmar patients with HCC, iron deposition might accelerate hepatocarcinogenesis. We believe that iron overload in human consumption through diet pathway such as by groundwater may be a potential and important risk factor for etiologic investigations of HCC. Iron can catalyze the production of oxygen radicals that may be proximate carcinogens. Moreover, iron may play a role of limiting nutrient for the growth and replication of cancer cells. Therefore, our study may provide the clue to investigate the causal role of exogenous iron from groundwater in the etiology of human liver carcinogenesis that cannot be ruled out and warrants further investigation by using Prussian blue staining or iron contents in biopsy liver specimens, or etc.

## Conclusions

The most interesting finding of this study is that the geographical variation in HCC mortality clearly depicted a marked coastal gradient pattern for men, whereas no such trend was observed for women. This study showed that HCC mortality is clustered in southwestern Taiwan and the association with the iron levels in groundwater in Taiwanese population warrant further investigation.

## Abbreviations

ASR: Age-standardized rate; GIS: Geographical Information System; HCC: Hepatocellular carcinoma, HBV, Hepatitis B virus; HCV: Hepatitis C virus; ICD: International Classification of Diseases.

## Competing interests

The authors declare that they have no competing interests.

## Authors’ contributions

YPL participated in the design, conducted the statistical analyses, interpreted the data, and drafted the manuscript. HJS and CCL supervised the study, assisted in data interpretation, and critically reviewed the manuscript. YHS, JYH, and CCH helped in conducting the study and revising the manuscript. PCK, CCP, YCC and SCC helped to manage and analyze the data. All authors read and approved the final manuscript.

## Pre-publication history

The pre-publication history for this paper can be accessed here:

http://www.biomedcentral.com/1471-2458/13/352/prepub
